# Leaky doors: Private captivity as a prominent source of bird introductions in Australia

**DOI:** 10.1371/journal.pone.0172851

**Published:** 2017-02-24

**Authors:** Miquel Vall-llosera, Phillip Cassey

**Affiliations:** School of Biological Sciences and Centre for Conservation Science and Technology (CCoST), The University of Adelaide, Adelaide, South Australia, Australia; Auburn University, UNITED STATES

## Abstract

The international pet trade is a major source of emerging invasive vertebrate species. We used online resources as a novel source of information for accidental bird escapes, and we investigated the factors that influence the frequency and distribution of bird escapes at a continental scale. We collected information on over 5,000 pet birds reported to be missing on animal websites during the last 15 years in Australia. We investigated whether variables linked to pet ownership successfully predicted bird escapes, and we assessed the potential distribution of these escapes. Most of the reported birds were parrots (> 90%), thus, we analysed factors associated with the frequency of parrot escapes. We found that bird escapes in Australia are much more frequent than previously acknowledged. Bird escapes were reported more frequently within, or around, large Australian capital cities. Socio-economic factors, such as the average personal income level of the community, and the level of human modification to the environment were the best predictors of bird escapes. Cheaper parrot species, Australian natives, and parrot species regarded as peaceful or playful were the most frequently reported escapees. Accidental introductions have been overlooked as an important source of animal incursions. Information on bird escapes is available online in many higher income countries and, in Australia, this is particularly apparent for parrot species. We believe that online resources may provide useful tools for passive surveillance for non-native pet species. Online surveillance will be particularly relevant for species that are highly reported, such as parrots, and species that are either valuable or highly commensal.

## Introduction

International wildlife trade moves millions of individual animals (of many thousands of species) around the world every year [1]. The pet industry, including the popular practice of keeping cage birds, is a large component of wildlife trade [2], and a significant source of new invasive vertebrate species [3–5]. However, the factors that influence which and how pet animals are released into the wild remain largely unstudied. Unlike the introduction of other wildlife commodities (e.g., fish stocking or game animal acclimatisation), the release of pet animals is an unfortunate consequence of the trade [4]. Pet species are either deliberately abandoned by their owners or can accidentally escape into the wild [6–8]. In some Asian countries, animals are also freed as part of religious practices [9,10]. Very few of these introduction events leave any identifiable records. This may be a substantial knowledge gap in contemporary invasion biology, which may be hindering biosecurity efforts for detecting new invasions and for preventing their impacts.

Former pets turned invasive species are capable of causing extinctions of native plants and animals and reducing biodiversity. They can threaten the organization and functioning of native communities through predation, competitive exclusion, disease transfer or hybridization [11–14]. However, not all released pets pose a high biosecurity risk. In order to become invasive, released pet birds must survive in the wild, establish a self-sustaining population and spread beyond the point of introduction [15,16], and indeed most introductions fail to do so [17]. Introduced bird populations are thought to be highly vulnerable to extinction by stochastic factors, given their generally small population sizes [18,19]. In addition, captive bred birds may have reduced invasive ability, through loss of genetic diversity or key natural behaviours [20–22].

Australia boasts some of the strictest biosecurity and quarantine regulations in the world, particularly in regard to the trade and importation of wildlife [23,24]. However, detections of new non-native vertebrates at large (in the wild) have continued to increase [25]. A recent review of vertebrate animal incursions in Australia reported over 227 bird incursion events detected by border and post-border biosecurity stakeholders during the 1990s [26]. Birds are also the most frequently released taxa (escapes and thefts) from public zoos [27]. Given that an estimated 13% of Australian households keep pet birds [28], private keeping may be the greatest existing source of bird introductions [27].

In the last decade, predominantly in higher income countries, different internet citizen initiatives have developed, particularly through social networks, with the aim of reuniting lost or stolen pets with their owners (e.g., https://www.parrotalert.com/; https://www.lostandfoundpetsaustralia.com). These resources provide relevant information on the identity and location of missing animals. We hypothesised that the distribution of bird escapes (i.e., unintentional pet bird losses) would be related to proxies of pet ownership; such as socio-economic characteristics of the community (Table 1). We used this information to predict the spatial distribution of the relative probability of bird escapes occurring across mainland Australia.

**Table 1:** Hypotheses related to the distribution of accidental bird releases.

Hypothesis	Prediction	Supporting evidence
Economic status	Higher income areas would be associated with a greater abundance of pets in private captivity and, consequently, more frequent escapes.	[5,29,30]
Population age structure: Elderly	Areas with a higher elderly population should have a greater frequency of escapes because: (i) a large proportion of the members of avicultural associations are people at the end of their active working lives and retirees; (ii) elderly people have more time and money to spend caring for pets; and (iii) elderly people, frequently living alone, show high level of attachment to pets.	[31–33]
Population age structure: Children	Families with children should also show an elevated propensity to pet ownership because children are particularly attached to pets.	[34–36]
Human population	Bird escapes should be more frequent in high human density areas.	[37,38]

## Materials and methods

### Data collection

We collated a unique dataset of accidental bird introductions in Australia using online reports of missing pet birds. We collected information on bird escapes from websites listed on the ‘Lost and Found’ section of the Australian Rescue and Rehoming Resource (ARRR) webpage (http://arrr.id.au/lostandfound.html; S1 Table), which contained reports for missing birds, for the period 1999–2013. From each report we extracted information on: (i) the identity of the species; (ii) the date and location of the report (including geographic coordinates); (iii) number of individuals missing (if known); and, (iv) the type of report (e.g., ‘Lost’, ‘Found’, or ‘Stolen’). We checked every report to assess the correct identification of the bird to the species level, or otherwise classified the bird as ‘Unknown’. We standardised the species scientific names according to Clements checklist of birds of the world Version 6.8 (http://www.birds.cornell.edu/clementschecklist/). Individual reports of bird escapes were cross-checked across all web pages to exclude duplicate reports of missing birds, and also in an endeavour to track the outcome of the escapees. On some occasions, birds reported initially as lost were later reported as found or even reunited with the owner. No personal data from the websites were recorded and all of the ‘Terms and Conditions’ of the individual sites were adhered to. The resulting data were analysed anonymously.

Economic data (average personal income level) and population age structure data were obtained from the Australian Bureau of Statistics. Previous research shows that non-native species richness is higher in areas of high human density [37]. Therefore, we included, as covariates in our analysis, measures of human population distribution such as: (i) Human Influence Index (HII), a measure showing direct human impact on ecosystems using eight measures of human presence [39]; and (ii) land use type for each Australian territory. HII was extracted from the Global Human Dataset of the Last of the Wild Project [39]. Land use data were obtained from the Australian Bureau of Agricultural and Resource Economics and Sciences website (see S1 Appendix for the complete details on data extraction, the list of references, and the corresponding links). All analyses were conducted in the R software environment for statistical computing and graphics version 3.03 [40]. Geographic data for all the predictors were available at different resolutions, therefore all of the datasets were resampled to the lowest resolution available, 5km grid cells, using the nearest neighbour method with the R-packages ‘raster’ [41] and ‘dismo’ [42]. This resolution matches the scale to which missing bird report data were collected (i.e., suburb level) and captures accurate spatial heterogeneity of where birds are most likely to escape captivity.

Over 90% of all individual birds reported ‘missing’ were parrots (Order: Psittaciformes) (see Results). Consequently, we analysed factors that were predicted to be associated with the frequency of unintentional parrot losses (Table 2). We collected information of the traded parrots in Australia from the bird price guides published by the Avicultural Societies from: (i) South Australia (seven editions: 2003, 2005, 2006, 2008, 2009, 2012, 2013 [43]); (ii) Victoria (three editions: 2011, 2012, 2013 [44]); and (iii) the dataset completed from the classified advertisements section of the two most popular aviculturist magazines in Australia (Australian Birdkeeper and Australian Aviculture [45,46]). We obtained information for 126 traded parrot species.

**Table 2:** Putative hypotheses related to the frequency of accidental parrot releases.

Hypothesis	Prediction	Supporting evidence
Propagule pressure	Cheaper species should have a greater chance of being released because they are more abundant in captivity, and because they are more likely to be kept under looser security measures.	[19,47,48]
Life history traits	Larger bodied and longer lived species should be less likely to escape because these species are usually more economically and emotionally valued and therefore more carefully kept.	[49–51]
Native status	Australian native parrots should be more likely to escape than non-natives, because natives are more abundant in domestic captivity, and because native species are less economically valued than non-natives.	[52,53]
Behavioural traits	Docile species should be less likely to escape because owners are more likely to become strongly bonded and care more about their welfare.	[54–56]

We collected information on the following pet/keeper related variables for each parrot species: (i) *Price* (AU$), information on abundance of parrots in captivity is not readily available in Australia because there is no registry of pet ownership for bird species. For this reason we used species price as a proxy for abundance. We predicted cheaper species should be more frequently released, because we assumed they are more abundant in private captivity. In a recent paper, we demonstrated that price and abundance in private captivity are inversely correlated for bird species in the domestic Australian trade [57]. Likewise, several lines of evidence point to the relationship between abundance in captivity and species price in the pet trade. Perception of rarity increases peoples’ willingness to pay high prices [58,59], therefore rarer species tend to be more expensive. The price for the escaped parrot species was obtained from the bird price guides. Price was estimated as the median, in Australian dollars, across all years and states, for a parent-reared pair comprising male and female of good quality and healthy condition. We considered only the price of the nominal subspecies because, for some species, there was considerable intraspecific variation in the price due to the presence in the trade of different subspecies or artificial breed colour mutations. Price was available for 107 species (84.9%); (ii) *Body mass* (g), body mass was estimated as the average body size of the adult male of the nominal subspecies. Body mass data was sourced from Dunning [60]. This information was available for 125 species (99.2%); (iii) *Longevity* (years), for each species, longevity was measured as the maximum number of years an animal is known to have survived in captivity [61–64]. Longevity was available for 88 (69.8%) species; (iv) *Docility*, we measured whether the species possessed (or not) attractive behavioural traits for the keeper as follows [57]. First, we reviewed information on parrot husbandry from the Birdcare webpage (http://birdcare.com.au/). The webpage extracts and summarizes information on bird husbandry from articles published in the Australian Aviculture and Australian Birdkeeper journals since 1947. For each of the parrot species we systematically checked the relevant information, searching for key words and terms that described the behavioural traits of the species in captivity related to docility. Second, we scored each species according to their descriptions as: (1) ‘Demanding’, if it was described as being harder to keep, for example, being shy, quiet, secretive, nervous, noisy, aggressive, prone to bite and/or requiring proper training or socialization; or (0) ‘Not demanding’, if described as active, playful, peaceful, and/or with the ability to learn to mimic and talk. This information was available for 116 species (92%); and (v) *Native status* of the species (native to Australia or not), the status of the species were defined according to the BirdLife Australia Working List of Australian Birds classification (http://birdlife.org.au/conservation/science/taxonomy; v1.1). The species were scored as ‘Non-native’ if their area of natural distribution did not include any part of the Australian territory, or ‘Native’ if otherwise. This information was available for all of the 126 parrot species. *Price* and *longevity* were log_e_ transformed and *body mass* was log_10_ transformed for further analysis. See S2 Table for the complete parrot dataset.

### Analysis

#### Spatial distribution of bird escapes

We used reports of missing birds for the period 2011–2013, including those in which the identity of the species was unknown. The majority of reports (> 85%) were from this period (see Results). We used generalised linear models (GLMs) to identify the factors associated with the spatial distribution of bird escapes in Australia. Escapes were recorded as a single presence data point. All reports provided information of the location, usually at the suburb level, and we used Google Maps to locate the geographic coordinates for each location. GLMs requires absence data but can be substituted by background data. Because no true-absence data are available for accidental escapes, a total of 10,000 random points were generated to be used as background data [65]. Given that the chance of escape, or detecting an escapee, in uninhabited areas (e.g., desert) is arguably close to zero, we used HII to place the random pseudo-absences within a buffer around human inhabited areas (including towns, cities and major roads) [66]. We used the lower scores of the HII to identify and exclude the areas of the Australian mainland without significant human impact (HII < 4; [39]). All areas outside the buffer were assumed to have a zero value, whereas all areas within the buffer were available for prediction. The buffer zone covered 61% of the surface of Australia. GLMs were fitted using the R package ‘dismo’. The occurrence of bird escapes (presence and pseudo-absence) were modelled with binomial variance and a logit link function. To avoid problems with model fitting, due to collinearity, we checked for correlated pairs of variables (Pearson’s r ≤ |0.7|). Collinearity amongst the variables was low (S3 Table). To balance model fit and predictive performance, the models were calculated using a 10-fold cross-validation procedure, which were run 50 times to ensure stable estimates of model evaluation statistics. A candidate set of models was built by including all possible combinations of explanatory variables to identify the models that provided the best support for the data, using the R-package ‘MuMIn’ [67]. The relative support for each model structure was assessed by ranking models based on Akaike Information Criterion corrected for small sample size (AIC_c_) and AIC_c_ weights (wAIC_c_) [68]. For the models with ΔAIC_c_ < 2 from the best model (lowest AIC_c_) for each run, we calculated the model-averaged coefficients for the estimates and standard deviations (S4 Table). The distribution of the values of the estimates, and standard deviations for the GLMs, is presented as a coefficient plot.

#### Determinants of parrot escapes

We used all reports of missing parrots, for the entire recorded period (1999–2013). Phylogenetic generalized least square models (PGLS, [69]) were fitted to analyse the relationship between the frequency of parrot escapes and the pet/keeper related variables using the ‘pgls’ function in the R package ‘caper’ [70,71]. We used PGLS models in order to account for the phylogenetic relatedness between the data points in the dependent variable, i.e. the species. This method addresses the issue of the ancestral relatedness by specifying a covariance matrix, which reflects the phylogenetic distances between the species. Each species is weighted according to the phylogenetic distance with the other species: the higher the correlation, the lower the weight given to that species. The PGLS model includes the estimated parameter Pagel’s lambda that controls the strength of the phylogenetic signal between the species [72]. Although escaping captivity is not a biological trait itself, i.e. shaped by evolution, we used PGLS because the propensity for escape is likely to be correlated with species traits, such as longevity and body mass, that are known to have a strong phylogenetic signal [27].

We based our phylogenetic informed analyses on the phylogenetic tree for bird species proposed by Jetz et al. [73]. The structure of this molecular-based tree is not known for certain, and so we incorporated uncertainty over the true phylogenetic relationship by repeating our analyses over a number of different phylogenetic trees for our species. One hundred avian phylogenetic trees were sampled at random online (http://birdtree.org), based on data from the complete avian phylogeny of Jetz et al. [73] and using the primary backbone tree of Hackett et al. [74]. These trees were used as alternative phylogenetic hypotheses for the evolutionary relatedness of the parrot species. For the analysis we excluded two taxa (*Barnardius barnadi* and *Pyrrhura roseifrons*), which were considered subspecies by Jetz et al. [73].

Parrot escapes were measured as a binary variable where a parrot species reported to the websites was either scored as ‘Reported’ (N = 65), or ‘Unreported’ (N = 61). Frequency of parrot escapes was measured as the number of reports lodged in missing animal websites for each species. We analysed the relationship between the parrot pet/keeper related variables and the frequency of parrot escapes fitting independent PGLS models for each variable. The distribution of the values of the estimates and standard deviation for all generated models are presented as coefficient plots.

## Results

### Bird escapes in Australia

**Fig 1:**
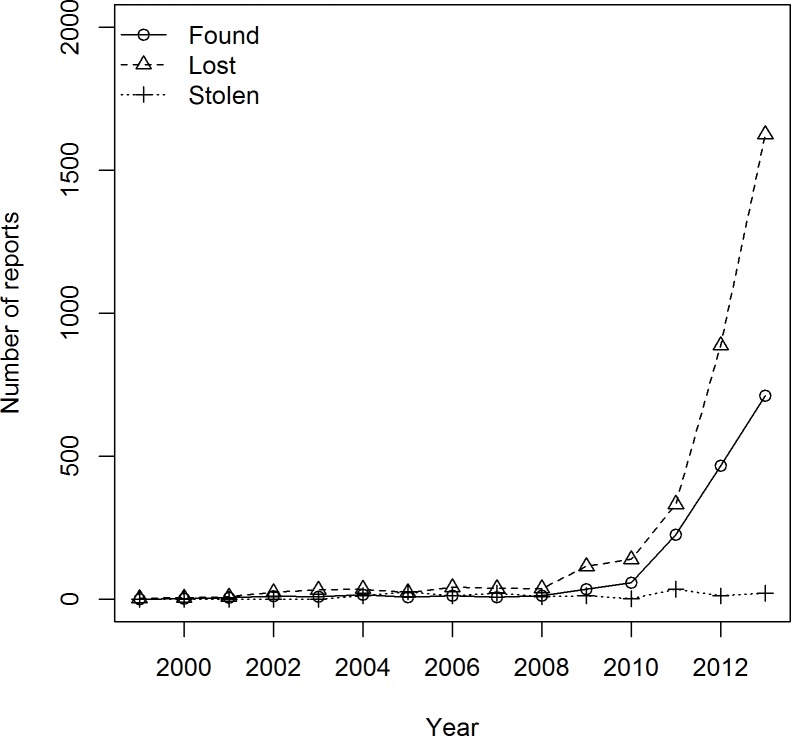
Frequency of reports of missing birds from private captivity during the period 1999–2013.

**Fig 2:**
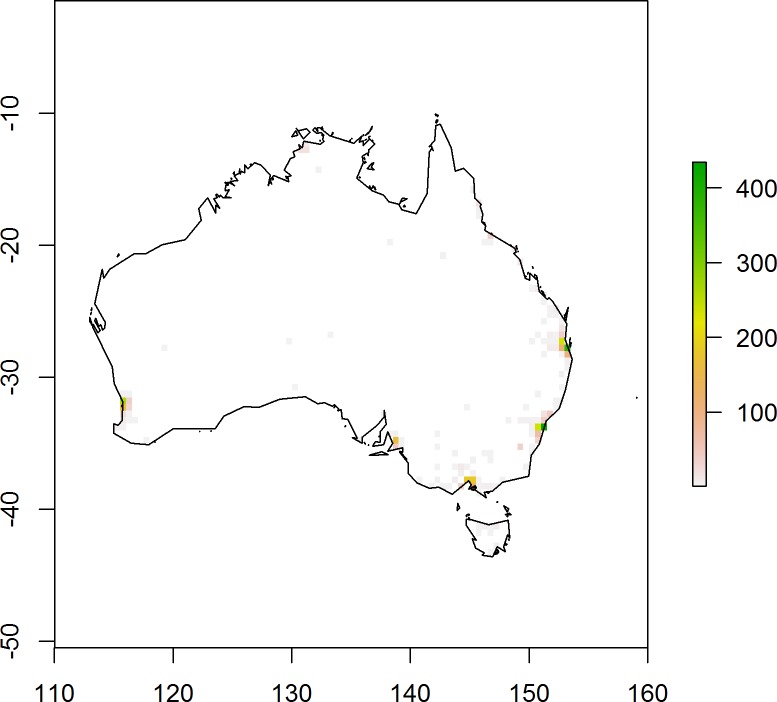
The distribution of bird escapes in Australia for the period 1999–2013. Presented as the number of missing bird reports per 60x60 km cell resolution. The Brisbane region accounted for 31.4% of all incursion events, followed by Sydney (29.9%) and Melbourne (16.1%).

We found reports for 5,139 bird escapes, corresponding to a total of 5,876 individuals from 91 bird species (six avian orders), for the period 1999–2013. For this period, the average number (± std err) of bird escapes was 120 ± 68 per month. Most reports of missing birds were from the period 2011–2013 (Fig 1). The majority of reports corresponded to lost birds (3,352 reports; 65.2%), followed by found birds (1,570 reports; 30.6%) and a small number of birds reported as stolen (217 reports; 4.2%). The average number of individuals reported per event was 1.14 ± 1.3 (N = 5,139). For stolen bird reports, the number of missing individuals reported was almost four times greater (3.8 ± 6; N = 217) than the average number reported for lost or found birds (1.04 ± 0.45; N = 5,103) (Fig 1). Missing birds were reported from urban or peri-urban areas, most of them concentrated within or around Australia’s seven major metropolitan regions, particularly along the east coast from Melbourne to Brisbane (Fig 2). This area accounted for 75% of all missing birds reported in Australia.

Native and non-native bird species were represented in similar proportions. During the study period, 42 non-native (45.7%) and 49 Australian native species (53.3%) were reported. However, the number of reports for native species was more than twice (3,366 escapes; 65.5%) the number of reports for non-native species (1,548 escapes; 32.5%) (Table 3). Parrots were the most reported avian order (Psittaciformes); including 72% of reports and 93% of all individuals (Table 3). The most frequently reported bird species was the Cockatiel *Nymphicus hollandicus*, a native species, with 28.9% of all reports, 2.5 times more frequent than the second, the Rose-ringed Parakeet *Psittacula krameri* (11.1% of all reports), which was the most frequently reported non-native species (Table 4).

**Table 3:** Total numbers of species, reports and individuals for birds reported to missing animal websites in Australia for the period 1999–2013.

Order	Common name	Species		Reports		Individuals	
		Native	Non-native	Native	Non-native	Native	Non-native
Psittaciformes	Parrots	39	27	3,351	1,467	3,669	1,787
Passeriformes	Finches	5	8	10	27	67	50
Galliformes	Wildfowl	2	3	2	20	4	22
Anseriformes	Waterfowl	1	2	1	10	1	15
Columbiformes	Pigeons & doves	1	2	1	24	1	26
Total		48	42	3,365	1,548	3,742	1,900

Not included are a total of 225 reports (228 individuals) of unidentified species, and one missing Emu *Dromaius novaehollandiae*.

**Table 4:** The five native and five non-native bird species most frequently reported to missing animal websites in Australia for the period 1999–2013.

Scientific name	Common name	Origin	Reports	Individuals
*Nymphicus hollandicus*	Cockatiel	Native	1,483	1,644
*Psittacula krameri*	Rose-ringed Parakeet	Non-native	570	637
*Eolophus roseicapilla*	Galah	Native	548	555
*Melopsittacus undulatus*	Budgerigar	Native	390	424
*Psittacula eupatria*	Alexandrine Parakeet	Non-native	390	416
*Eclectus roratus*	Eclectus Parrot	Native	299	305
*Trichoglossus haematodus*	Rainbow Lorikeet	Native	166	172
*Pyrrhura molinae*	Green-cheeked Conure	Non-native	127	139
*Myiopsitta monachus*	Monk Parakeet	Non-native	124	128
*Aratinga solstitialis*	Sun Conure	Non-native	76	81

The remaining species (n = 81) had less than 150 reports combined.

### Spatial distribution of bird escapes

**Fig 3:**
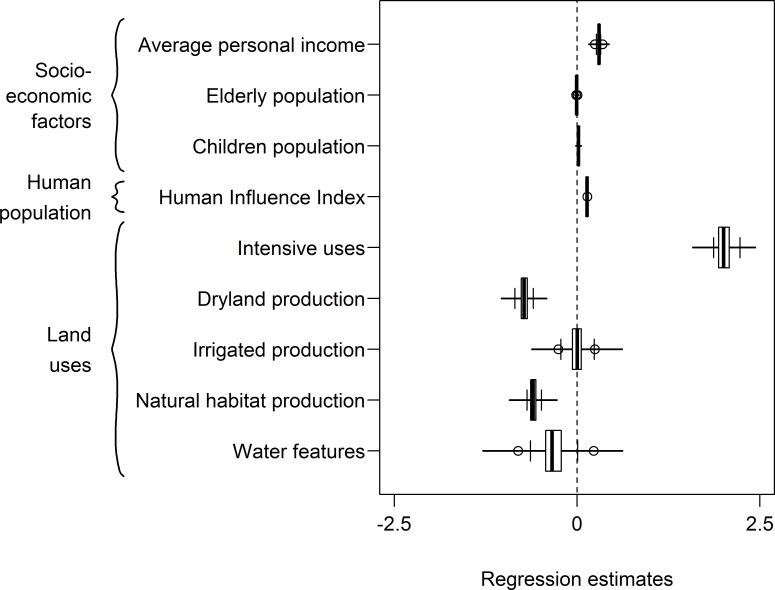
Coefficient plot of the generalised linear model for the predictors of the spatial distribution of bird escapes in Australia. Positive regression estimates represent higher frequency of escapes, and vice versa.

The performance of the model for predicting the spatial distribution of bird escapes was very high (average AIC [5th; 95th percentile] = 2,444.6[2,382.4; 2,501.7]; compared to the null model, AIC = 17,523). The distribution of bird escape reports was related to socio-economic factors of the community. The areas with higher average personal income level observed a greater frequency of reports. We found that bird escapes were more likely to occur in areas with higher levels of human modification to the environment. Bird escape reports were related to higher levels of HII and strongly positively related to intensive land uses (Fig 3 and S5 Table).

### Determinants of parrot escapes

**Fig 4:**
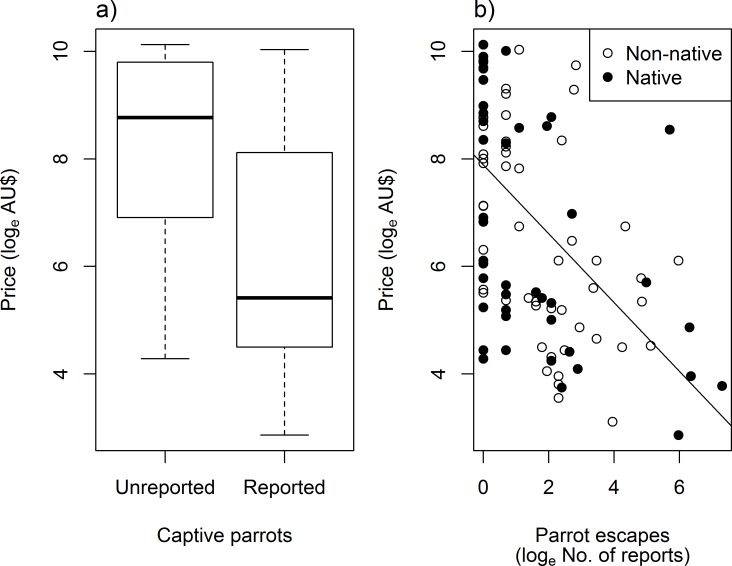
The relationship between species price in the Australian domestic trade. (a) Whether or not a parrot species has escaped captivity, and (b) the frequency of parrot escapes (non-natives and natives) in Australia.

**Fig 5:**
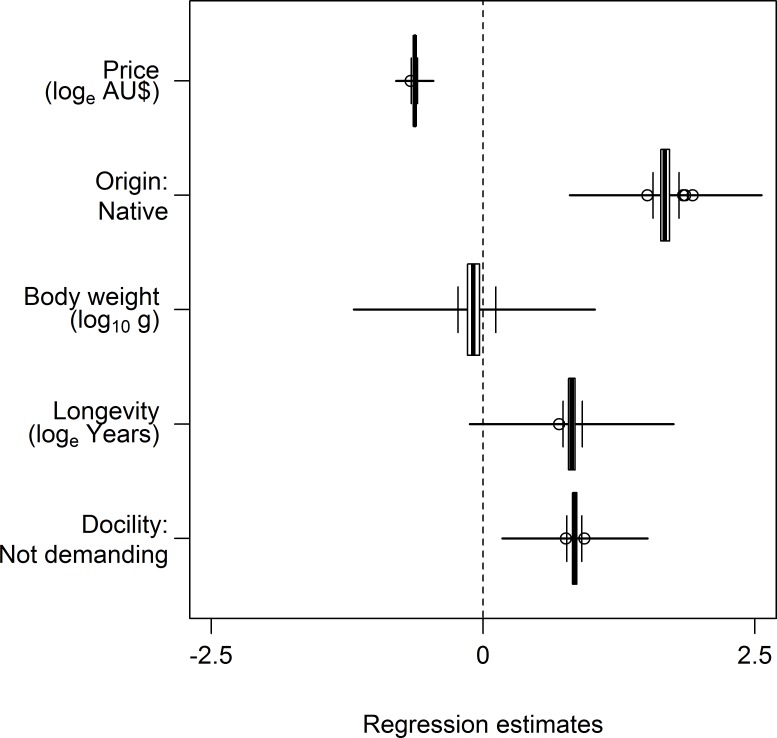
Coefficient plot for the model averaged phylogenetic logistic regression analyses for determinants of parrot escapes. Positive regression estimates represent higher frequency of introductions, and vice versa.

For the parrot species reported on missing animal websites (compared with all parrot species present in the Australian domestic bird trade) we found evidence for a relatively strong phylogenetic signal in the tendency for parrots to be reported as lost, found, or stolen (Estimated median lambda [5^st^, 95^th^ percentiles] = 0.62 [0.55, 0.69]). We found a strong association between parrot escapes and the species price. Parrots reported on missing animal web sites were cheaper than species that have never been reported as escapees (Fig 4A). Similarly, parrot species that escaped most frequently were cheaper than species that less frequently escaped, or that did not escape at all (median t-value [5^st^, 95^th^ percentiles] = -7.21 [-7.44, -7.02], df = 105, P < 0.001; Fig 4B). Parrot escapes were positively associated with the geographical origin and with the behaviour of the species as a pet (Fig 5). The parrot species escaping most frequently were mostly natives and had docile temperaments, i.e. species regarded as playful and peaceful (Fig 5 and S6 Table).

## Discussion

Pet trade is the dominant transport pathway for non-native bird species globally [5], and it is estimated that four million live birds are traded annually [75]. As a consequence, captivity is a major source of introduced bird species. In Australia, we have found that non-native bird introductions occur much more frequently than previously reported [25]. The difference is not in the number of species (38 in Henderson & Bomford [25] *c*.*f*. 41 in this study), but in the number of introduction events and individuals: seven times more than previously recorded. When we included escaped natives, the numbers of reported species doubled and the number of introduction events and individuals increased by a factor of 22, with respect to previous estimates. The role of the pet keeping in the introduction pathway of vertebrates (particularly birds) has been largely overlooked [52,76]. The main reason is that animals reported to online public platforms are not generally recorded in the scientific literature or in the records of Australian biosecurity agencies. The difference in the proportion of reports for lost and found birds indicates that most birds that go missing are never found again, once in the wild. Indeed, detecting escaped birds appears to have a low probability. We suggest that only using reports of non-native birds seen at large might not be the best method for detecting emergent pests and quantifying incursions.

The expansion of the internet has changed how information is exchanged worldwide [77]. These developments have produced new biosecurity risks [78,79], but also novel surveillance opportunities. In the case of escaped pet birds, the owners are able to communicate their losses with the local community of pet keepers in the hope of being reunited with their pets, and these records are available for tracking introduction events. The evidence collected from these online resources has revealed that hundreds of captive pet birds are unintentionally released from private households every month across the continent. We have no evidence to believe that the dramatic increase after 2010 is due to more birds escaping. Instead, we suggest that this is a likely consequence of the growing popularity, and participation, in social media and internet usage.

We found, as expected, that the distribution of bird escapes is strongly linked to the distribution of human population in Australia. Missing bird reports came from urban and peri-urban areas [37,38]. We found that the distribution of escaped birds is related to socio-economic factors of the community. Reported bird escapes were more frequent in areas with higher incomes. This supports our hypothesis that personal wealth could be related to higher pet ownership [30]. Moreover, most areas with high average income correspond to highly inhabited areas. However, it appears that greater personal income is not related to the economic value of escaped species, because the species most frequently reported were, on average, less economically valued species.

The number of escaped bird species reported is low compared to the number of bird species known to be present in private captivity in Australia. Parrot species constitute only 35.9% of the traded bird species in Australia [57], yet lost parrots made up the majority of reports on the missing animal websites. We have no evidence to suggest that parrots escape more than other species. In public institutions (i.e. zoos), previous studies have shown that parrots were not more likely to escape, than other species [27]. However, we suggest that parrots are more likely to be reported missing than other cagebird species. We do not know whether this is because parrots are more valued (sentimentally) than other bird groups, or because their relatively large body-size, bright colours and behaviour are considered more likely to be successfully found and returned. Alternately, finches and softbills (see S2 Appendix for definitions) are underrepresented in missing animal reports despite being abundant and widespread in private captivity in Australia [57]. We suggest that missing individuals from these bird groups are probably less likely to be reported as lost, even for those with high economical value, because of lower chances of recovering the escaped birds. Regardless, if other bird species are escaping as frequently as parrots, and not being reported, then the pet trade in Australia has a very leaky aviary door [4].

Parrots species that were more frequently reported as escaped were cheaper, on average, than parrot species that were less frequently reported, or that were never reported as escaped. This result supports our hypothesis that less economically valued (more abundant) species are more likely to be deliberately or accidentally released [57]. Previous research has found that popular species in the pet trade are introduced more frequently than rarer species [47,80,81]. Given that the complete confinement of birds in captivity can never be guaranteed, the more widely held a species is the greater the likelihood that an escape will occur [82]. In addition, because abundance in private captivity for most bird species in Australia is unknown; we suggest that price can be used as a proxy to predict the captive pet bird species which have higher chances of being released, or escaping [57]. Species price can also be related to abundance in the wild. Recent research on the illegal bird trade and parrot conservation showed that the species that are more abundant and accessible in the wild tend to be the cheaper, while rare species tend to be more expensive [83–85]. Perception of rarity is a factor driving extinctions of wild-caught traded species, particularly in countries where poaching for the pet trade is poorly controlled [83,86,87].

Australian native species were the most frequently escaped parrots. In a previous study we showed that native parrot species are almost as abundant in private captivity as non-natives [57]. Recent studies also revealed that easily available species are more common in the bird markets [83,85], and Australia has a great diversity and abundance of native parrot species [64]. This situation contrasts with bird trade in other Western countries like Europe [88], where most traded bird species are non-native, but it is more similar to Asian countries [9,10]. Native species are not always introduced in their natural range within the country [89], and this is particularly apparent for range restricted species. There is plenty of evidence of native bird species becoming pests and causing impacts just as significant as non-native species [90–92]. Some studies have suggested, that the so called ‘domestic exotics’ should be considered as biosecurity risks, alongside non-native species, outside their natural distribution [89].

The very high frequency of bird escapes suggests that the establishment of new non-native populations will eventually occur [19]. However, despite the high rate of reported bird escapes in Australia, there is no evidence of the widespread risk of new ‘pet’ invaders. A small proportion of the escaped birds (30.6%) are found afterwards but most escapees disappear. Birds are generally kept in low numbers and, consequently, reported escapes involve a few individuals, and most often only a single individual (> 95% cases). It is possible that these escaped birds do not survive at large long enough to find mates and reproduce. In particular, captive birds may have lost some of the skills necessary to survive in nature [20,22]. For example, parrots intended to be sold as pets are usually socialized with humans in order for them to easily bond with their owners [51]. Indeed, we found that more docile parrot species were more frequently reported on the missing animal websites as escapees. Owners may bond more easily with well-behaved species, therefore increasing their motivation for reporting the loss. Most recovered parrots are found in the vicinity of the release point, usually in bird feeders. The only circumstances in which the probability of establishment would be higher would be in the case of enough individuals released in one location, over a sufficient short period of time [93]; for example, repeated leaks from an unsecured aviary.

Our study has revealed that Australia has a genuine problem within domestic bird-keeping and onshore biosecurity reporting. The domestic pet trade constitutes an important pathway for the introduction of birds in Australia, and is of much greater risk than escapes from public holdings [27]. However, unlike species kept in zoos and aquariums, measures for preventing the escapes and thefts from private holdings are non-existent or unenforced.

Banning the import of potential invasive wildlife has often been suggested as a preventive measure to mitigate the impact of invasive species [94,95]. Given that Australia already has a very reduced list of bird species that can be legally imported [24], we do not think further restrictions to the imports would affect the size of non-native species populations in captivity in Australia. Current domestic regulations on keeping non-native wildlife appear to be useful in preventing the introduction of some extreme risk species [96,97]. However, while regulating domestic breeding for these species (e.g. Rose-ringed Parakeet) may help to reduce population sizes available for escape, these kind of measures would certainly be very unpopular among the pet trade sector. International regulations to wildlife trade, such as CITES [98], have also been offered as a solution to stop over-harvesting of threatened species [99,100]. In a previous paper, we found that trade regulation at a national level had a greater effect on species price (and hence on abundance in captivity) in Australia than international bans [57]. Finally, native Australian parrot wild populations are protected from international and domestic wildlife trade by biosecurity [23] and state and territory regulations [101]. In general, native populations of Australian parrots are healthy and those species facing conservation issues have problems unrelated to harvesting for trade [102,103].

We suggest that bird reports from missing animal websites could be used as a passive surveillance system to monitor accidental bird introductions and to detect areas of high and frequent non-independent escapes. We advocate for intervention in situations that pose higher risk of establishment, such as when breeding activity in the wild is detected. We strongly recommend that introductions of the most frequently escaped non-native species should be monitored to prevent any future risk of invasion. For example, the Rose-ringed Parakeet is the most frequently escaping non-native bird species in Australia (260 releases only in 2013). The parakeet is considered an extreme risk species [26], and has previously been known to breed at large [104]. The risk posed by the parakeet is clearly worthy of additional study [66].

Internet surveillance is a novel tool, based on public participation, but it also has its limitations. For example, there is great variability in individual people’s internet use, which we would expect to affect reporting. Globally, there is uneven access to the Internet, particularly in low-income countries, where many people lack access and access may be restricted to large cities (http://data.worldbank.org/indicator/IT.NET.USER.P2?view=map). Finally, we detected that there is a component of reporting bias regarding escaped species and there is still the likelihood that introductions will occur unnoticed.

Even with these limitations, we believe that reports to missing animal websites are a useful record of accidental pet bird introductions. Escaped parrots, in particular, may pose a greater risk to become new pest species. A better registry of bird keeping, and monitoring of escapes, is required to track incursions and to inform biosecurity agencies of the risk posed by the escapes of species present in the animal pet trade.

## Supporting information

S1 Table Complete list of consulted webpages. List obtained from the Lost and Found section of the Australian Rescue and Rehoming Resource webpage. All webpages were last accessed in December 2013.
(DOCX)

S2 Table Complete parrot dataset. NA means that the data was not available.
(DOCX)

S3 Table Pearson’s r correlation coefficient for predictors of bird escapes in Australia (N = 12,766). Only continuous variables.
(DOCX)

S4 Table Summary of the components of the ΔAIC_c_ < 2 models. Components were calculated using a 10-fold cross-validation and 50 runs. For each run, 32 models (combinations of the 5 variables) were compared.Variable codes are: 1. Human Influence Index; 2. Land uses; 3. Average personal income; 4. Elderly population; and 5. Children population.
(DOCX)

S5 Table Model coefficients (median estimates, [5th, 95th percentiles]) in GLMs for predictors of spatial distribution of bird escapes in Australia. Coefficients were calculated using a 10-fold cross-validation and 50 runs.
(DOCX)

S6 Table Model coefficients (median estimates, [5th, 95th percentiles]) in PGLS models for determinants of parrot escapes. Coefficients were calculated over 100 randomly selected likely phylogenetic trees. 'Estimate' means the 'lambda' value of the PGLS model.
(DOCX)

S1 Appendix Predictors of the spatial distribution of accidental bird escapes in Australia. (DOCX)

S2 Appendix Definition of the softbill and finch avicultural groups. (DOCX)
