# Supplementary material for: Leaky doors: Private captivity as a prominent source of bird introductions in Australia
Source: PLoS One. 2017 Feb 24;12(2):e0172851. doi: 10.1371/journal.pone.0172851 (PMC5325556; doi:10.1371/journal.pone.0172851)
Supplement: S1 Table — List obtained from the Lost and Found section of the Australian Rescue and Rehoming Resource webpage. All webpages were last accessed in December 2013. (DOCX) [file pone.0172851.s001.docx]

| **Website name** | **Link** |
| --- | --- |
| All Creatures Lost and Found Inc | http://allcreatureslostandfound.com.au/type/lost-bird/ |
| Australian Pet Link | http://www.petlink.com.au/Lost_and_Found/ |
| Find Losts Pets | http://letsfindyourpet.com.au/search_lost_pets_database.php?state=Select+a+State&specie=3&sex=0&submit.x=33&submit.y=10&submit=submit |
| Flealess Market's Lost Pets International | http://www.flealess.org/lostpets/australia.html |
| Free classifieds in Australia | http://www.olx.com.au/q/parrot/c-413 |
| Lost & Found Pets Newcastle | http://lostpetsnewcastle.com.au/index.php?option=com_adsmanager&page=show_category&catid=1&text_search=&order=0&expand=0&Itemid=0 |
| Lost and Found | http://www.lostandfound.com/search/lost/advanced |
| Lost and Found Pets Australia | http://www.lostandfoundpetsaustralia.com/SearchResults.aspx?SearchType=LostAnimals |
| Lost Pet Finders | http://lostpetfinders.com.au/pets?filter=all&q= |
| Parrot Society of Australia Inc | http://www.parrotsociety.org.au/index.php?mod=Dynamic&id=66 |
| parrotalert.com | http://www.parrotalert.com/search-directory.php |
| Pet Search | http://www.petsearch.com.au/ShowPets.asp?CurrentPage=4&Type=13 |
| Queensland Lost Pet Register | http://www.rspcaqld.org.au/LostAndFound/SearchLostPets |
| The Pet Directory | http://www.petdirectory.com.au/?page=lostandfound_pets_dogs_cats_birds |
| Top End Lost and Found | http://telaf.wordpress.com/?s=ringneck |
