# Supplementary material for: Leaky doors: Private captivity as a prominent source of bird introductions in Australia
Source: PLoS One. 2017 Feb 24;12(2):e0172851. doi: 10.1371/journal.pone.0172851 (PMC5325556; doi:10.1371/journal.pone.0172851)
Supplement: S2 Table — NA means that the data was not available. (DOCX) [file pone.0172851.s002.docx]

| **Scientific name** | **Common name** | **Escapes** | **Reports** | **Native status** | **Price** | **Body mass** | **Longevity** | **Docility** |
| --- | --- | --- | --- | --- | --- | --- | --- | --- |
| *Probosciger aterrimus* | Palm Cockatoo | Unreported | 0 | Native | NA | 841 | 56.33 | Demanding |
| *Calyptorhynchus baudinii* | White-tailed Black-Cockatoo | Reported | 1 | Native | 4000 | 620 | 47 | Demanding |
| *Calyptorhynchus latirostris* | Slender-billed Black-Cockatoo | Reported | 1 | Native | 3750 | 612 | NA | Demanding |
| *Calyptorhynchus funereus* | Yellow-tailed Black-Cockatoo | Reported | 2 | Native | 5325 | 740 | 41 | Demanding |
| *Calyptorhynchus banksii* | Red-tailed Black-Cockatoo | Reported | 10 | Native | 4200 | 775 | 45.5 | Not demanding |
| *Calyptorhynchus lathami* | Glossy Black-Cockatoo | Unreported | 0 | Native | 16000 | 437 | NA | Demanding |
| *Callocephalon fimbriatum* | Gang-gang Cockatoo | Reported | 1 | Native | 2600 | 256 | 27.5 | Demanding |
| *Eolophus roseicapilla* | Galah | Reported | 548 | Native | 130 | 332 | 40 | Not demanding |
| *Lophochroa leadbeateri* | Pink Cockatoo | Reported | 31 | Native | 450 | 376 | 63.58 | Demanding |
| *Cacatua galerita* | Sulphur-crested Cockatoo | Reported | 145 | Native | 300 | 790 | 57 | Demanding |
| *Cacatua moluccensis* | Salmon-crested Cockatoo | Unreported | 0 | Exotic | NA | 835 | 65.75 | Demanding |
| *Cacatua alba* | White Cockatoo | Unreported | 0 | Exotic | NA | 570 | 26.92 | Demanding |
| *Cacatua sanguinea* | Little Corella | Reported | 31 | Native | 105 | 562 | 46.92 | Not demanding |
| *Cacatua pastinator* | Western Corella | Reported | 7 | Native | 150 | 750 | 26.33 | Not demanding |
| *Cacatua tenuirostris* | Long-billed Corella | Reported | 18 | Native | 130 | 588 | 40 | Not demanding |
| *Nymphicus hollandicus* | Cockatiel | Reported | 1483 | Native | 43.75 | 92.2 | 35 | Not demanding |
| *Chalcopsitta atra* | Black Lory | Unreported | 0 | Exotic | 8000 | 195 | 17.5 | Not demanding |
| *Chalcopsitta sintillata* | Yellow-streaked Lory | Unreported | 0 | Exotic | NA | 190 | 19.67 | NA |
| *Eos histrio* | Red-and-blue Lory | Unreported | 0 | Exotic | 13000 | 167.5 | 15.17 | Demanding |
| *Eos squamata* | Violet-necked Lory | Unreported | 0 | Exotic | NA | 110 | NA | Not demanding |
| *Eos bornea* | Red Lory | Unreported | 0 | Exotic | 3250 | 156 | 24.67 | Not demanding |
| *Eos reticulata* | Blue-streaked Lory | Unreported | 0 | Exotic | NA | 150 | 16.58 | Not demanding |
| *Pseudeos fuscata* | Dusky Lory | Unreported | 0 | Exotic | 2750 | 149 | 13.42 | Demanding |
| *Trichoglossus ornatus* | Ornate Lorikeet | Unreported | 0 | Exotic | 8000 | 110 | 19.42 | Not demanding |
| *Trichoglossus haematodus* | Rainbow Lorikeet | Reported | 166 | Native | 92.5 | 120 | 16.08 | Not demanding |
| *Trichoglossus chlorolepidotus* | Scaly-breasted Lorikeet | Reported | 17 | Native | 60 | 87.1 | NA | Not demanding |
| *Psitteuteles versicolor* | Varied Lorikeet | Unreported | 0 | Native | 262.5 | 53.5 | NA | Not demanding |
| *Psitteuteles goldiei* | Goldie's Lorikeet | Unreported | 0 | Exotic | 8000 | 58.7 | 11 | Not demanding |
| *Lorius garrulus* | Chattering Lory | Unreported | 0 | Exotic | 4250 | 143 | 26.58 | Not demanding |
| *Lorius domicella* | Purple-naped Lory | Unreported | 0 | Exotic | NA | 182 | 26 | Not demanding |
| *Lorius lory* | Black-capped Lory | Reported | 1 | Exotic | 4125 | 183 | NA | Not demanding |
| *Lorius chlorocercus* | Yellow-bibbed Lory | Unreported | 0 | Exotic | 7000 | 187 | NA | Not demanding |
| *Glossopsitta concinna* | Musk Lorikeet | Reported | 5 | Native | 90 | 76.1 | 11.42 | Not demanding |
| *Glossopsitta pusilla* | Little Lorikeet | Unreported | 0 | Native | 188.75 | 39.4 | NA | Not demanding |
| *Glossopsitta porphyrocephala* | Purple-crowned Lorikeet | Unreported | 0 | Native | 247.5 | 44.7 | 12 | Demanding |
| *Charmosyna toxopei* | Blue-fronted Lorikeet | Unreported | 0 | Exotic | NA | NA | NA | NA |
| *Cyclopsitta diophthalma* | Double-eyed Fig-Parrot | Unreported | 0 | Native | 3000 | 48.9 | 6.42 | Demanding |
| *Eclectus roratus* | Eclectus Parrot | Reported | 299 | Native | 5150 | 561 | 28.5 | Not demanding |
| *Alisterus scapularis* | Australian King-Parrot | Reported | 28 | Native | 270 | 222 | 26.58 | Not demanding |
| *Aprosmictus erythropterus* | Red-winged Parrot | Reported | 4 | Native | 250 | 136 | 24.42 | NA |
| *Polytelis swainsonii* | Superb Parrot | Reported | 10 | Native | 180 | 153 | 15.08 | Not demanding |
| *Polytelis anthopeplus* | Regent Parrot | Reported | 7 | Native | 205 | 170 | 13.75 | Not demanding |
| *Polytelis alexandrae* | Princess Parrot | Reported | 69 | Native | 90 | 96 | 23.92 | Not demanding |
| *Purpureicephalus spurius* | Red-capped Parrot | Reported | 1 | Native | 240 | 116 | 15.33 | Demanding |
| *Barnardius zonarius* | Port Lincoln Parrot | Reported | 7 | Native | 185 | 140 | 17.92 | NA |
| *Platycercus caledonicus* | Green Rosella | Unreported | 0 | Native | 925 | 149 | NA | Not demanding |
| *Platycercus elegans* | Crimson Rosella | Reported | 3 | Native | 225 | 140 | 15.5 | Not demanding |
| *Platycercus venustus* | Northern Rosella | Unreported | 0 | Native | 450 | 92.6 | 19.08 | Not demanding |
| *Platycercus adscitus* | Pale-headed Rosella | Reported | 4 | Native | 210 | 111 | NA | NA |
| *Platycercus eximius* | Eastern Rosella | Reported | 7 | Native | 70 | 104 | 27.42 | Not demanding |
| *Platycercus icterotis* | Western Rosella | Reported | 4 | Native | 195 | 65.4 | 13.17 | Not demanding |
| *Northiella haematogaster* | Bluebonnet | Reported | 1 | Native | 180 | 85.5 | 15.33 | NA |
| *Psephotus haematonotus* | Red-rumped Parrot | Reported | 9 | Native | 52.5 | 61.4 | 12.25 | Not demanding |
| *Psephotus varius* | Mulga Parrot | Reported | 1 | Native | 160 | 61.5 | 11.75 | Demanding |
| *Psephotus dissimilis* | Hooded Parrot | Reported | 1 | Native | 215 | 45.8 | 18 | Not demanding |
| *Psephotus chrysopterygius* | Golden-shouldered Parrot | Reported | 1 | Native | 285 | 46.8 | NA | NA |
| *Cyanoramphus novaezelandiae* | Red-fronted Parakeet | Reported | 11 | Exotic | 85 | 82.1 | 12.42 | Not demanding |
| *Cyanoramphus auriceps* | Yellow-fronted Parakeet | Unreported | 0 | Exotic | 85 | 51.2 | 9.33 | Not demanding |
| *Neophema bourkii* | Bourke's Parrot | Reported | 9 | Native | 35 | 44.4 | 12.58 | Not demanding |
| *Neophema chrysostoma* | Blue-winged Parrot | Reported | 1 | Native | 85 | 46.1 | 21 | NA |
| *Neophema elegans* | Elegant Parrot | Reported | 6 | Native | 57.5 | 43.4 | NA | Not demanding |
| *Neophema petrophila* | Rock Parrot | Unreported | 0 | Native | 325 | 53.2 | NA | Demanding |
| *Neophema pulchella* | Turquoise Parrot | Reported | 7 | Native | 75 | 42.7 | 21 | Not demanding |
| *Neophema splendida* | Scarlet-chested Parrot | Reported | 13 | Native | 82.5 | 38.6 | 14 | NA |
| *Lathamus discolor* | Swift Parrot | Unreported | 0 | Native | 1243.75 | 64.7 | NA | NA |
| *Melopsittacus undulatus* | Budgerigar | Reported | 390 | Native | 17.5 | 29.2 | NA | Not demanding |
| *Psittacus erithacus* | Gray Parrot | Reported | 15 | Exotic | 10800 | 333 | 49.67 | Demanding |
| *Poicephalus gulielmi* | Red-fronted Parrot | Unreported | 0 | Exotic | NA | 290 | 21 | Not demanding |
| *Poicephalus senegalus* | Senegal Parrot | Reported | 1 | Exotic | 10005 | 147 | 40 | Demanding |
| *Poicephalus meyeri* | Meyer's Parrot | Unreported | 0 | Exotic | 20000 | 120 | 34.17 | Not demanding |
| *Poicephalus rufiventris* | Red-bellied Parrot | Unreported | 0 | Exotic | 20000 | 120 | 33.42 | Not demanding |
| *Agapornis canus* | Gray-headed Lovebird | Unreported | 0 | Exotic | NA | 30 | NA | Not demanding |
| *Agapornis roseicollis* | Rosy-faced Lovebird | Reported | 51 | Exotic | 22.5 | 54.5 | 12.58 | Not demanding |
| *Agapornis fischeri* | Fischer's Lovebird | Reported | 10 | Exotic | 42.5 | 48.3 | NA | Not demanding |
| *Agapornis personatus* | Yellow-collared Lovebird | Reported | 9 | Exotic | 45 | 49 | NA | Not demanding |
| *Agapornis lilianae* | Lilian's Lovebird | Unreported | 0 | Exotic | 72.5 | 37.3 | 19.17 | Demanding |
| *Psittacula eupatria* | Alexandrine Parakeet | Reported | 390 | Exotic | 450 | 214 | 30 | Not demanding |
| *Psittacula krameri* | Rose-ringed Parakeet | Reported | 570 | Exotic | 52.5 | 126 | 34 | Not demanding |
| *Psittacula himalayana* | Slaty-headed Parakeet | Unreported | 0 | Exotic | 550 | 125 | NA | Not demanding |
| *Psittacula cyanocephala* | Plum-headed Parakeet | Reported | 5 | Exotic | 225 | 66 | 18.75 | Not demanding |
| *Psittacula columboides* | Malabar Parakeet | Reported | 1 | Exotic | 3350 | 85.5 | 11.17 | Not demanding |
| *Psittacula derbiana* | Derbyan Parakeet | Unreported | 0 | Exotic | 4250 | 251 | NA | Demanding |
| *Psittacula alexandri* | Red-breasted Parakeet | Reported | 14 | Exotic | 650 | 147 | 23.33 | Demanding |
| *Anodorhynchus hyacinthinus* | Hyacinth Macaw | Unreported | 0 | Exotic | NA | 1331 | 38.83 | Demanding |
| *Ara ararauna* | Blue-and-yellow Macaw | Reported | 16 | Exotic | 17000 | 1125 | 43 | Demanding |
| *Ara macao* | Scarlet Macaw | Reported | 1 | Exotic | 22250 | 1015 | 33 | Not demanding |
| *Ara chloropterus* | Red-and-green Macaw | Reported | 2 | Exotic | 22750 | 1214 | 50.08 | Not demanding |
| *Ara rubrogenys* | Red-fronted Macaw | Unreported | 0 | Exotic | 25000 | 468 | 22.5 | Not demanding |
| *Ara severus* | Chestnut-fronted Macaw | Unreported | 0 | Exotic | 25000 | 343 | 28.5 | Demanding |
| *Primolius maracana* | Blue-winged Macaw | Unreported | 0 | Exotic | 25000 | 256 | 31 | Demanding |
| *Primolius auricollis* | Yellow-collared Macaw | Unreported | 0 | Exotic | 25000 | 245 | NA | Demanding |
| *Diopsittaca nobilis* | Red-shouldered Macaw | Reported | 6 | Exotic | 5500 | 156 | 22.92 | Not demanding |
| *Aratinga acuticaudata* | Blue-crowned Parakeet | Unreported | 0 | Exotic | 13000 | 171 | 31 | Not demanding |
| *Guarouba guarouba* | Golden Parakeet | Unreported | 0 | Exotic | 25000 | 194 | 23.25 | Not demanding |
| *Aratinga solstitialis* | Sun Parakeet | Reported | 76 | Exotic | 850 | 120.5 | NA | Not demanding |
| *Aratinga jandaya* | Jandaya Parakeet | Reported | 14 | Exotic | 1075 | 89.4 | NA | Not demanding |
| *Aratinga auricapillus* | Golden-capped Parakeet | Reported | 1 | Exotic | 6750 | 130 | 20.92 | Not demanding |
| *Aratinga weddellii* | Dusky-headed Parakeet | Unreported | 0 | Exotic | NA | 108 | NA | Not demanding |
| *Aratinga aurea* | Peach-fronted Parakeet | Unreported | 0 | Exotic | 5500 | 86.5 | NA | Not demanding |
| *Aratinga pertinax* | Brown-throated Parakeet | Unreported | 0 | Exotic | NA | 84 | NA | Not demanding |
| *Nandayus nenday* | Nanday Parakeet | Reported | 9 | Exotic | 450 | 128 | 18.67 | Demanding |
| *Cyanoliseus patagonus* | Burrowing Parakeet | Unreported | 0 | Exotic | 1000 | 286 | 19.5 | Demanding |
| *Pyrrhura cruentata* | Ochre-marked Parakeet | Reported | 2 | Exotic | 2500 | 90 | NA | Not demanding |
| *Pyrrhura frontalis* | Maroon-bellied Parakeet | Unreported | 0 | Exotic | 425 | 88.5 | NA | Not demanding |
| *Pyrrhura lepida* | Pearly Parakeet | Reported | 2 | Exotic | 850 | 75 | NA | Not demanding |
| *Pyrrhura perlata* | Crimson-bellied Parakeet | Reported | 1 | Exotic | 4000 | 75 | 14.33 | Not demanding |
| *Pyrrhura molinae* | Green-cheeked Parakeet | Reported | 127 | Exotic | 210 | 77.1 | NA | Not demanding |
| *Pyrrhura picta* | Painted Parakeet | Unreported | 0 | Exotic | 18000 | 62.1 | NA | Not demanding |
| *Pyrrhura leucotis* | Maroon-faced Parakeet | Unreported | 0 | Exotic | 20000 | 51.5 | 18.5 | Not demanding |
| *Pyrrhura egregia* | Fiery-shouldered Parakeet | Unreported | 0 | Exotic | 6000 | 66.3 | NA | Not demanding |
| *Pyrrhura rupicola* | Black-capped Parakeet | Unreported | 0 | Exotic | 1250 | 75 | NA | Not demanding |
| *Pyrrhura rhodocephala* | Rose-headed Parakeet | Unreported | 0 | Exotic | NA | 80 | NA | Not demanding |
| *Myiopsitta monachus* | Monk Parakeet | Reported | 124 | Exotic | 325 | 120 | 22.08 | Demanding |
| *Bolborhynchus lineola* | Barred Parakeet | Unreported | 0 | Exotic | NA | 56.2 | NA | Not demanding |
| *Pionites melanocephalus* | Black-headed Parrot | Unreported | 0 | Exotic | 6375 | 157 | 22.58 | Not demanding |
| *Pionites leucogaster* | White-bellied Parrot | Unreported | 0 | Exotic | 18500 | 155 | 26 | Not demanding |
| *Pionus menstruus* | Blue-headed Parrot | Unreported | 0 | Exotic | NA | 251 | 20.58 | Not demanding |
| *Amazona leucocephala* | Cuban Parrot | Unreported | 0 | Exotic | 25000 | 227 | NA | Demanding |
| *Amazona albifrons* | White-fronted Parrot | Unreported | 0 | Exotic | 16250 | 206 | 25.33 | Demanding |
| *Amazona finschi* | Lilac-crowned Parrot | Unreported | 0 | Exotic | NA | 297 | 25.75 | Not demanding |
| *Amazona autumnalis* | Red-lored Parrot | Unreported | 0 | Exotic | NA | 416 | 27 | Demanding |
| *Amazona aestiva* | Turquoise-fronted Parrot | Reported | 7 | Exotic | 6500 | 451 | 49 | Not demanding |
| *Amazona ochrocephala* | Yellow-crowned Parrot | Unreported | 0 | Exotic | 6500 | 440 | 56 | Demanding |
| *Amazona amazonica* | Orange-winged Parrot | Unreported | 0 | Exotic | NA | 370 | 30 | Demanding |
| *Amazona oratrix* | Yellow-headed Parrot | Reported | 1 | Exotic | 11000 | 517 | NA | Demanding |
| *Amazona auropalliata* | Yellow-naped Parrot | Unreported | 0 | Exotic | 13000 | 440 | 49 | Demanding |
