# Supplementary material for: Leaky doors: Private captivity as a prominent source of bird introductions in Australia
Source: PLoS One. 2017 Feb 24;12(2):e0172851. doi: 10.1371/journal.pone.0172851 (PMC5325556; doi:10.1371/journal.pone.0172851)
Supplement: S3 Table — Only continuous variables. (DOCX) [file pone.0172851.s003.docx]

|  | **Human population density** | **Average personal income level** | **Elderly population** | **Children population** |
| --- | --- | --- | --- | --- |
| Human Influence Index | 0.73 | 0.24 | 0.25 | -0.28 |
| Human population  density |  | 0.31 | 0.10 | -0.31 |
| Average personal  income level |  |  | -0.30 | -0.21 |
| Elderly  population |  |  |  | -0.30 |
