# Supplementary material for: Leaky doors: Private captivity as a prominent source of bird introductions in Australia
Source: PLoS One. 2017 Feb 24;12(2):e0172851. doi: 10.1371/journal.pone.0172851 (PMC5325556; doi:10.1371/journal.pone.0172851)
Supplement: S4 Table — Components were calculated using a 10-fold cross-validation and 50 runs. For each run, 32 models (combinations of the 5 variables) were compared.Variable codes are: 1. Human Influence Index; 2. Land uses; 3. Average personal income; 4. Elderly population; and 5. Children population. (DOCX) [file pone.0172851.s004.docx]

| **Run** | **Variables** | **df** | **LogLik** | **AIC_c_** | **ΔAIC_c_** | **wAIC_c_** |
| --- | --- | --- | --- | --- | --- | --- |
| 1 | 123 | 8 | -1203.8 | 2423.6 | 0.0 | 0.5 |
|  | 1235 | 9 | -1203.3 | 2424.7 | 1.1 | 0.3 |
|  | 1234 | 9 | -1203.8 | 2425.5 | 2.0 | 0.2 |
| 2 | 123 | 8 | -1229.5 | 2474.9 | 0.0 | 0.5 |
|  | 1235 | 9 | -1229.0 | 2476.0 | 1.0 | 0.3 |
|  | 1234 | 9 | -1229.4 | 2476.8 | 1.9 | 0.2 |
| 3 | 123 | 8 | -1211.9 | 2439.9 | 0.0 | 0.5 |
|  | 1235 | 9 | -1211.4 | 2440.7 | 0.9 | 0.3 |
|  | 1234 | 9 | -1211.8 | 2441.7 | 1.8 | 0.2 |
| 4 | 123 | 8 | -1170.6 | 2357.2 | 0.0 | 0.5 |
|  | 1235 | 9 | -1170.4 | 2358.8 | 1.6 | 0.2 |
|  | 1234 | 9 | -1170.4 | 2358.9 | 1.7 | 0.2 |
| 5 | 123 | 8 | -1210.6 | 2437.3 | 0.0 | 0.5 |
|  | 1235 | 9 | -1210.1 | 2438.3 | 1.0 | 0.3 |
|  | 1234 | 9 | -1210.4 | 2438.7 | 1.5 | 0.2 |
| 6 | 123 | 8 | -1221.7 | 2459.4 | 0.0 | 0.4 |
|  | 1235 | 9 | -1220.9 | 2459.8 | 0.4 | 0.4 |
|  | 1234 | 9 | -1221.6 | 2461.2 | 1.8 | 0.2 |
| 7 | 123 | 8 | -1229.6 | 2475.2 | 0.0 | 0.5 |
|  | 1235 | 9 | -1229.2 | 2476.3 | 1.2 | 0.3 |
|  | 1234 | 9 | -1229.5 | 2477.1 | 1.9 | 0.2 |
| 8 | 123 | 8 | -1202.7 | 2421.3 | 0.0 | 0.5 |
|  | 1235 | 9 | -1202.1 | 2422.2 | 0.9 | 0.3 |
|  | 1234 | 9 | -1202.6 | 2423.1 | 1.8 | 0.2 |
| 9 | 123 | 8 | -1212.5 | 2441.0 | 0.0 | 0.5 |
|  | 1235 | 9 | -1212.2 | 2442.4 | 1.4 | 0.3 |
|  | 1234 | 9 | -1212.5 | 2443.0 | 1.9 | 0.2 |
| 10 | 123 | 8 | -1183.3 | 2382.6 | 0.0 | 0.5 |
|  | 1235 | 9 | -1182.6 | 2383.2 | 0.6 | 0.3 |
|  | 1234 | 9 | -1183.1 | 2384.1 | 1.6 | 0.2 |
| 11 | 123 | 8 | -1203.9 | 2423.8 | 0.0 | 0.5 |
|  | 1235 | 9 | -1203.7 | 2425.5 | 1.7 | 0.2 |
|  | 1234 | 9 | -1203.8 | 2425.6 | 1.7 | 0.2 |
| 12 | 123 | 8 | -1229.5 | 2474.9 | 0.0 | 0.5 |
|  | 1235 | 9 | -1228.7 | 2475.4 | 0.5 | 0.4 |
|  | 1234 | 9 | -1229.3 | 2476.7 | 1.7 | 0.2 |
| 13 | 123 | 8 | -1226.8 | 2469.5 | 0.0 | 0.5 |
|  | 1235 | 9 | -1226.4 | 2470.8 | 1.3 | 0.3 |
|  | 1234 | 9 | -1226.7 | 2471.4 | 1.9 | 0.2 |
| 14 | 123 | 8 | -1257.9 | 2531.8 | 0.0 | 0.5 |
|  | 1235 | 9 | -1257.4 | 2532.9 | 1.2 | 0.3 |
|  | 1234 | 9 | -1257.8 | 2533.7 | 1.9 | 0.2 |
| 15 | 123 | 8 | -1233.8 | 2483.7 | 0.0 | 0.5 |
|  | 1235 | 9 | -1233.0 | 2484.1 | 0.4 | 0.4 |
|  | 1234 | 9 | -1233.7 | 2485.5 | 1.8 | 0.2 |
| 16 | 123 | 8 | -1226.9 | 2469.9 | 0.0 | 0.5 |
|  | 1235 | 9 | -1226.2 | 2470.4 | 0.5 | 0.4 |
|  | 1234 | 9 | -1226.9 | 2471.7 | 1.9 | 0.2 |
| 17 | 123 | 8 | -1206.1 | 2428.2 | 0.0 | 0.5 |
|  | 1235 | 9 | -1205.8 | 2429.5 | 1.4 | 0.3 |
|  | 1234 | 9 | -1206.0 | 2430.1 | 2.0 | 0.2 |
| 18 | 1235 | 9 | -1199.3 | 2416.7 | 0.0 | 0.4 |
|  | 123 | 8 | -1200.6 | 2417.3 | 0.6 | 0.3 |
|  | 1234 | 9 | -1200.3 | 2418.7 | 2.0 | 0.1 |
|  | 12345 | 10 | -1199.3 | 2418.7 | 2.0 | 0.1 |
| 19 | 123 | 8 | -1190.8 | 2397.6 | 0.0 | 0.5 |
|  | 1235 | 9 | -1190.3 | 2398.6 | 1.0 | 0.3 |
|  | 1234 | 9 | -1190.6 | 2399.2 | 1.6 | 0.2 |
| 20 | 123 | 8 | -1209.9 | 2435.8 | 0.0 | 0.5 |
|  | 1235 | 9 | -1209.3 | 2436.6 | 0.7 | 0.3 |
|  | 1234 | 9 | -1209.6 | 2437.3 | 1.5 | 0.2 |
| 21 | 123 | 8 | -1214.2 | 2444.4 | 0.0 | 0.5 |
|  | 1235 | 9 | -1213.7 | 2445.5 | 1.1 | 0.3 |
|  | 1234 | 9 | -1214.2 | 2446.3 | 2.0 | 0.2 |
| 22 | 123 | 8 | -1213.5 | 2443.1 | 0.0 | 0.5 |
|  | 1235 | 9 | -1213.2 | 2444.4 | 1.3 | 0.3 |
|  | 1234 | 9 | -1213.5 | 2445.1 | 2.0 | 0.2 |
| 23 | 123 | 8 | -1218.7 | 2453.4 | 0.0 | 0.4 |
|  | 1235 | 9 | -1218.1 | 2454.2 | 0.8 | 0.3 |
|  | 1234 | 9 | -1218.2 | 2454.4 | 0.9 | 0.3 |
| 24 | 123 | 8 | -1232.9 | 2481.7 | 0.0 | 0.6 |
|  | 1234 | 9 | -1232.3 | 2482.7 | 1.0 | 0.4 |
| 25 | 1235 | 9 | -1193.5 | 2405.1 | 0.0 | 0.4 |
|  | 123 | 8 | -1195.0 | 2405.9 | 0.9 | 0.3 |
|  | 1234 | 9 | -1194.5 | 2407.0 | 1.9 | 0.2 |
|  | 12345 | 10 | -1193.5 | 2407.0 | 2.0 | 0.2 |
| 26 | 123 | 8 | -1201.7 | 2419.3 | 0.0 | 0.4 |
|  | 1235 | 9 | -1200.8 | 2419.5 | 0.2 | 0.4 |
|  | 1234 | 9 | -1201.4 | 2420.9 | 1.6 | 0.2 |
| 27 | 1234 | 9 | -1216.8 | 2451.7 | 0.0 | 0.6 |
|  | 123 | 8 | -1218.2 | 2452.5 | 0.8 | 0.4 |
| 28 | 123 | 8 | -1241.4 | 2498.9 | 0.0 | 0.5 |
|  | 1235 | 9 | -1240.8 | 2499.6 | 0.7 | 0.3 |
|  | 1234 | 9 | -1241.4 | 2500.8 | 1.9 | 0.2 |
| 29 | 123 | 8 | -1221.1 | 2458.3 | 0.0 | 0.5 |
|  | 1235 | 9 | -1220.5 | 2458.9 | 0.6 | 0.3 |
|  | 1234 | 9 | -1221.1 | 2460.3 | 2.0 | 0.2 |
| 30 | 123 | 8 | -1182.4 | 2380.9 | 0.0 | 0.5 |
|  | 1235 | 9 | -1181.7 | 2381.5 | 0.6 | 0.3 |
|  | 1234 | 9 | -1182.4 | 2382.8 | 2.0 | 0.2 |
| 31 | 123 | 8 | -1208.5 | 2433.0 | 0.0 | 0.5 |
|  | 1235 | 9 | -1208.1 | 2434.3 | 1.3 | 0.3 |
|  | 1234 | 9 | -1208.5 | 2435.0 | 2.0 | 0.2 |
| 32 | 123 | 8 | -1232.1 | 2480.1 | 0.0 | 0.5 |
|  | 1235 | 9 | -1231.5 | 2481.0 | 0.8 | 0.3 |
|  | 1234 | 9 | -1232.0 | 2482.1 | 2.0 | 0.2 |
| 33 | 123 | 8 | -1191.4 | 2398.8 | 0.0 | 0.5 |
|  | 1235 | 9 | -1191.2 | 2400.4 | 1.6 | 0.3 |
|  | 1234 | 9 | -1191.4 | 2400.8 | 2.0 | 0.2 |
| 34 | 123 | 8 | -1203.5 | 2423.0 | 0.0 | 0.5 |
|  | 1235 | 9 | -1203.3 | 2424.6 | 1.5 | 0.3 |
|  | 1234 | 9 | -1203.5 | 2425.0 | 1.9 | 0.2 |
| 35 | 123 | 8 | -1201.6 | 2419.3 | 0.0 | 0.4 |
|  | 1235 | 9 | -1200.8 | 2419.6 | 0.3 | 0.3 |
|  | 12345 | 10 | -1200.5 | 2421.0 | 1.7 | 0.2 |
|  | 1234 | 9 | -1201.6 | 2421.3 | 2.0 | 0.1 |
| 36 | 123 | 8 | -1180.6 | 2377.3 | 0.0 | 0.5 |
|  | 1235 | 9 | -1180.4 | 2378.8 | 1.5 | 0.3 |
|  | 1234 | 9 | -1180.6 | 2379.3 | 2.0 | 0.2 |
| 37 | 123 | 8 | -1220.9 | 2457.8 | 0.0 | 0.5 |
|  | 1235 | 9 | -1220.6 | 2459.3 | 1.5 | 0.3 |
|  | 1234 | 9 | -1220.8 | 2459.6 | 1.8 | 0.2 |
| 38 | 123 | 8 | -1225.5 | 2467.1 | 0.0 | 0.6 |
|  | 1234 | 9 | -1224.8 | 2467.7 | 0.6 | 0.4 |
| 39 | 123 | 8 | -1212.0 | 2440.1 | 0.0 | 0.5 |
|  | 1235 | 9 | -1211.4 | 2440.9 | 0.8 | 0.3 |
|  | 1234 | 9 | -1211.9 | 2441.9 | 1.8 | 0.2 |
| 40 | 123 | 8 | -1226.0 | 2468.1 | 0.0 | 0.4 |
|  | 1235 | 9 | -1225.2 | 2468.3 | 0.3 | 0.4 |
|  | 1234 | 9 | -1226.0 | 2470.0 | 1.9 | 0.2 |
| 41 | 123 | 8 | -1204.6 | 2425.2 | 0.0 | 0.4 |
|  | 1234 | 9 | -1203.9 | 2425.9 | 0.7 | 0.3 |
|  | 1235 | 9 | -1204.2 | 2426.3 | 1.2 | 0.2 |
| 42 | 123 | 8 | -1243.2 | 2502.3 | 0.0 | 0.5 |
|  | 1235 | 9 | -1242.6 | 2503.1 | 0.8 | 0.3 |
|  | 1234 | 9 | -1243.2 | 2504.3 | 2.0 | 0.2 |
| 43 | 1235 | 9 | -1232.9 | 2483.8 | 0.0 | 0.5 |
|  | 123 | 8 | -1234.1 | 2484.1 | 0.4 | 0.4 |
|  | 1234 | 9 | -1233.8 | 2485.6 | 1.9 | 0.2 |
| 44 | 123 | 8 | -1206.0 | 2428.0 | 0.0 | 0.5 |
|  | 1235 | 9 | -1205.5 | 2428.9 | 0.9 | 0.3 |
|  | 1234 | 9 | -1205.8 | 2429.5 | 1.5 | 0.2 |
| 45 | 123 | 8 | -1252.6 | 2521.3 | 0.0 | 0.5 |
|  | 1235 | 9 | -1252.2 | 2522.4 | 1.0 | 0.3 |
|  | 1234 | 9 | -1252.6 | 2523.2 | 1.9 | 0.2 |
| 46 | 123 | 8 | -1197.6 | 2411.3 | 0.0 | 0.6 |
|  | 1235 | 9 | -1197.6 | 2413.1 | 1.8 | 0.2 |
|  | 1234 | 9 | -1197.6 | 2413.2 | 1.9 | 0.2 |
| 47 | 123 | 8 | -1233.0 | 2481.9 | 0.0 | 0.5 |
|  | 1235 | 9 | -1232.3 | 2482.5 | 0.6 | 0.3 |
|  | 1234 | 9 | -1232.9 | 2483.8 | 1.8 | 0.2 |
| 48 | 1235 | 9 | -1202.0 | 2421.9 | 0.0 | 0.4 |
|  | 123 | 8 | -1203.0 | 2422.0 | 0.0 | 0.4 |
|  | 12345 | 10 | -1201.9 | 2423.8 | 1.9 | 0.1 |
|  | 1234 | 9 | -1202.9 | 2423.9 | 1.9 | 0.1 |
| 49 | 123 | 8 | -1205.6 | 2427.2 | 0.0 | 0.5 |
|  | 1235 | 9 | -1205.0 | 2428.0 | 0.8 | 0.3 |
|  | 1234 | 9 | -1205.5 | 2429.0 | 1.9 | 0.2 |
| 50 | 123 | 8 | -1210.3 | 2436.7 | 0.0 | 0.5 |
|  | 1235 | 9 | -1210.0 | 2437.9 | 1.3 | 0.3 |
|  | 1234 | 9 | -1210.1 | 2438.2 | 1.6 | 0.2 |
