# Supplementary material for: Leaky doors: Private captivity as a prominent source of bird introductions in Australia
Source: PLoS One. 2017 Feb 24;12(2):e0172851. doi: 10.1371/journal.pone.0172851 (PMC5325556; doi:10.1371/journal.pone.0172851)
Supplement: S5 Table — Coefficients were calculated using a 10-fold cross-validation and 50 runs. (DOCX) [file pone.0172851.s005.docx]

| **Predictors** |  | **Estimate** | **Std. Error** | **z value** | **p value** |
| --- | --- | --- | --- | --- | --- |
| Socio-economic factors | Average personal income | 0.33 [0.31, 0.34] | 0.07 [0.07, 0.08] | 4.39 [4.08, 4.67] | 0.00 [0.00, 0.00] |
| Population age structure | Elderly population | -0.01 [-0.01, 0.00] | 0.00 [0.01, 0.01] | 0.66 [0.21, 1.07] | 0.52 [0.28, 0.83] |
|  | Children population | 0.01 [0.01, 0.02] | 0.02 [0.02, 0.02] | 0.68 [0.39, 0.99] | 0.51 [0.32, 0.70] |
| Human impact | Human Influence Index | 0.14 [0.14, 0.14] | 0.00 [0.00, 0.00] | 34.91 [34.65, 35.19] | 0.00 [0.00, 0.00] |
| Land uses | Intensive uses | 2.14 [2.01, 2.27] | 0.23 [0.22, 0.23] | 9.39 [8.96, 9.81] | 0.00 [0.00, 0.00] |
|  | Dryland production | -0.69 [-0.76, -0.61] | 0.16 [0.16, 0.16] | 4.31 [3.80, 4.75] | 0.00 [0.00, 0.00] |
|  | Irrigated production | 0.05 [-0.11, 0.19] | 0.32 [0.31, 0.32] | 0.28 [0.02, 0.62] | 0.79 [0.53, 0.98] |
|  | Natural environments production | -0.56 [-0.63, -0.48] | 0.17 [0.17, 0.17] | 3.29 [2.81, 3.70] | 0.00 [0.00, 0.01] |
|  | Water features | 0.02 [-0.23, 0.20] | 0.46 [0.44, 0.49] | 0.25 [0.07, 0.55] | 0.80 [0.59, 0.95] |
