# Supplementary material for: Leaky doors: Private captivity as a prominent source of bird introductions in Australia
Source: PLoS One. 2017 Feb 24;12(2):e0172851. doi: 10.1371/journal.pone.0172851 (PMC5325556; doi:10.1371/journal.pone.0172851)
Supplement: S6 Table — Coefficients were calculated over 100 randomly selected likely phylogenetic trees. 'Estimate' means the 'lambda' value of the PGLS model. (DOCX) [file pone.0172851.s006.docx]

| **Predictors** | **Estimate** | **Std. Error** | **t value** | **p value** |
| --- | --- | --- | --- | --- |
| Price (log_e_ AU$) | -0.63 [-0.65, -0.61] | 0.09 [0.09, 0.09] | -7.21 [-7.44, -7.02] | 0.00 [0.00, 0.00] |
| Origin: Native | 1.68 [1.59, 1.79] | 0.45 [0.44, 0.47] | 3.73 [3.57, 3.96] | 0.00 [0.00, 0.00] |
| Body mass (log_10_ gr) | -0.04 [-0.08, 0.02] | 0.25 [0.23, 0.26] | -0.15 [-0.35, 0.07] | 0.87 [0.73, 0.99] |
| Longevity (log_e_ Year) | 0.82 [0.75, 0.88] | 0.48 [0.47, 0.49] | 1.71 [1.56, 1.83] | 0.09 [0.07, 0.12] |
| Docility: Not demanding | 0.85 [0.80, 0.90] | 0.34 [0.33, 0.35] | 2.48 [2.35, 2.65] | 0.01 [0.01, 0.02] |
