# Supplementary material for: Leaky doors: Private captivity as a prominent source of bird introductions in Australia
Source: PLoS One. 2017 Feb 24;12(2):e0172851. doi: 10.1371/journal.pone.0172851 (PMC5325556; doi:10.1371/journal.pone.0172851)
Supplement: S1 Appendix — (DOCX) [file pone.0172851.s007.docx]

**S1 Appendix**

**Predictors of the spatial distribution of accidental bird escapes in Australia.**

We obtained data on average personal income level and population age structure from the Australian Bureau of Statistics. All information was collected at the level of Local Government Areas. Average personal income, in tens of thousands AU$, was sourced from the Australian Tax Office (ATO). The ATO presents regional data on the number of income earners and amounts they received from a number of different sources. From the ‘Estimates of Personal Income for Small Areas Time Series’ 2005-06 to 2010-11 document, we calculated the mean value for the ‘Average Total Income’ from all sources (excluding Government pensions and allowances) for that period of time (<http://www.abs.gov.au/ausstats/abs@.nsf/mf/6524.0.55.002>; last accessed December 2013). Data on the population age structure was extracted from the ABS document ‘Population Estimates by Age and Sex’ for Regions of Australia (<http://www.abs.gov.au/Ausstats/abs@.nsf/mf/3235.0>; last accessed December 2013). Data was calculated from ‘Population Estimates by Age’ for the year 2011. The Elderly population variable was calculated as the proportion of the estimated resident population (i.e. including males and females) over 65 years old. The Children population variable was calculated as the proportion of the estimated resident population under 14 years old.

Human impact was measured using data from the Global Human Footprint Dataset of the Last of the Wild Project ((1) Last of the Wild Project, Version 2, 2005 (LWP-2): Global Human Footprint Dataset (Geographic); <http://sedac.ciesin.columbia.edu/data/set/wildareas-v2-human-footprint-geographic>; last accessed March 2014). The Global Human Footprint Dataset of the Last of the Wild Project is the Human Influence Index (HII) normalized by biome and realm. The HII is a global dataset of 1km grid cells, created from nine global data layers covering human population pressure (population density), human land use and infrastructure (built-up areas, night-time lights, land use/land cover), and human access (coastlines, roads, railroads, navigable rivers). The data is available in geographic coordinate system at 30 arc-second grid cell size (~1km grid cells). The dataset is produced by the Wildlife Conservation Society (WCS).

Land use data was obtained from Land Use of Australia map (Version 4, 2005-2006; <http://data.daff.gov.au/anrdl/metadata_files/pa_luav4g9abl07811a00.xml>, last accessed March 2014), available at the Australian Bureau of Agricultural and Resource Economics and Sciences (ABARES) website. The map is supplied as a grid with 0.01 degree cell size (~1.5km grid cells).The types of agricultural land uses are based on the 2005-06 agricultural census data collected by the Australian Bureau of Statistics (ABS). The land is classified according to the Australian Land Use and Management Classification (ALUMC), Version 7 (<http://www.daff.gov.au/abares/aclump/pages/land-use/alum-classification-version-7-may-2010/alum-classification-version-7-may-2010.aspx>). We used the first level of classification, including a total of 6 different classes of land: conservation and natural environments, production from dry and irrigated agriculture and plantations, and from relatively natural habitats, intensive uses and water features. Intensive uses include farming production, residential infrastructure, services, utilities and transport.

**References**

1. Sanderson EW, Jaiteh M, Levy MA, Redford KH, Wannebo AV, Woolmer G. The Human Footprint and the Last of the Wild. Bioscience. 2002;52(10):891–904.
