# Supplementary material for: Leaky doors: Private captivity as a prominent source of bird introductions in Australia
Source: PLoS One. 2017 Feb 24;12(2):e0172851. doi: 10.1371/journal.pone.0172851 (PMC5325556; doi:10.1371/journal.pone.0172851)
Supplement: S2 Appendix — (DOCX) [file pone.0172851.s008.docx]

**S2 Appendix**

**Definition of the softbill and finch avicultural groups.**

The domestic trade classifies birds in groups not according to their phylogenetic relatedness, but that roughly match their taxonomic categories, such as finches or softbills (1–6). Softbills is the name given by aviculturists to a taxonomic heterogeneous group of cage birds characterized by a frugivorous or insectivorous diet. This group includes avian orders such as Coraciiformes (including kingfishers and bee-eaters) and Charadriformes (including dotterels and lapwings), as well as families of non granivorous passerines such as Turdidae (thrushes), Pycnonotidae (bulbuls) and Corvidae (including jays and magpies). Finches are, also, a paraphyletic group of birds that includes granivorous passerines of the families Estrildidae, Emberizidae, Fringillidae, Passeridae, Ploceidae, Thraupidae and Viduidae.

**References**

1. The United Bird Societies of South Australia Inc. Bird Price Guide. 1st to 7th edition. 2003-2013.

2. The Avicultural Society of Australia Inc. Guide to Bird Prices. 2011-2012 to 2013-2014. Australian Aviculture. 2012-2014.

3. The Avicultural Society of Australia Inc. Member’s Notices Birds for Sale & Wanted to Buy. Australian Aviculture. 2011-2013.

4. The Queensland Finch Society Inc. Finch Price Guide. 2012 to 2014. Finch News. 2012-2014.

5. Fitt G, Pace D. National Finch Census 2011- An Analysis. Aust Birdkeep. 2011;24(11):735–737.

6. Fitt G, Pace D. 3rd National Finch Census 2014 – An Analysis. National Finch and Softbill Association; 2014. 13 p.
